# Suicidal ideation and suicide attempts among Tunisian adolescents: prevalence and associated factors

**DOI:** 10.11604/pamj.2019.34.105.19920

**Published:** 2019-10-22

**Authors:** Asma Guedria-Tekari, Sonia Missaoui, Wassim Kalai, Naoufel Gaddour, Lotfi Gaha

**Affiliations:** 1 Department of Psychiatry, Fattouma Bourguiba University Hospital, Monastir, Tunisia; 2 University of Monastir, Monastir, Tunisia; 3 Service de Pédopsychiatrie, CISSS de Lanaudière, Québec, Canada

**Keywords:** Suicide, adolescent, suicidal behavior, suicidal ideation, suicide attempts, suicide risk factors

## Abstract

**Introduction:**

Studies directed on suicidal behavior in North African adolescents are rare. This study was conducted to estimate the prevalence of suicidal thoughts and attempts among high school students in Tunisia and to determine factors associated with this suicidal behavior.

**Methods:**

This is an analytical cross-sectional study composed of a population containing 821 high school students obtained through cluster sampling. The participants completed a pre-established form related to socio-demographic characteristics and anxiety symptoms, the Suicide Behavior Questionnaire-Revised, the Beck depression scale, and the Rosenberg self-esteem scale.

**Results:**

The mean age of the adolescents was 17.7±0.97 years. Prevalence of suicidal behavior was 26.9% for brief passing suicidal thoughts, 9.6% for serious suicidal thoughts, and 7.3% for suicide attempts. Six determining factors of suicidal behavior were found: female gender (OR=2.56 (1.32-4.95); p= 0.005), personal history of depression (OR=2.29 (1.38-3.80); p=0.001), tobacco smoking (OR=3.59 (1.61-8.01); p=0.002), current depression symptoms (OR=5.50 (2.14-14.11); p<0.001), history of non-suicidal self-injurious behavior (OR= 3.16 (2.05-4.86); p<0.001), and low self-esteem (OR=2.74 (1.71-4.38); p<0.001).

**Conclusion:**

Suicidal ideation and attempts are frequent among Tunisian adolescents and represent a serious public health problem. An urgent national prevention program is required.

## Introduction

Suicidal behavior in adolescents is steadily increasing worldwide over the last few years [1]. The adolescence is a period of transition characterized by various physical and psychological transformations, which can be the source of an enormous psychological distress [2]. It represents a period of higher vulnerability to everyday difficulties leading to impulsive and/or unpredictable behavior such as suicidal behavior [3]. Beside this internal adolescents-specific vulnerability to suicidal behavior, most investigators emphasize the impact of the existence of a dysfunction within the family, school or social environment. This external dimension seems to be of greater importance according to recent research [4,5]. Although suicidal behavior is particularly frequent among adolescents suffering from mental disorders including depression, schizophrenia, bipolar disorder, and substance abuse, during adolescence this behavior is not necessarily linked to an underlying pathology or a real desire for death [6]. Suicide is the result of a whole coordinated suicidal process. At the beginning, suicidal ideas that are usually intermittent appear, and intensify later to become permanent and invasive. These ideas lead to the establishment of a suicidal plan and the accomplishment of that process lead subsequently to death. Whenever the outcome of the act is not fatal, it is defined as a suicide attempt which represents any act performed under the same conditions as suicide but does not lead to death [7,8]. In young people, this suicidal process can take place very quickly over time giving it an impulsive character [9].

In recent decades, the prevalence of adolescent suicide has continued to increase, making this cause of death a major public health problem in different parts of the world in terms of loss of life [10]. As for other suicidal behavior, the prevalence among this range of age remains variable. The prevalence of suicide attempts is estimated to be around 10% [11], whereas the prevalence of suicidal ideation can reach up to 50% [12]. While most studies about suicidal behavior in adolescents have been conducted in high-income countries, Rukundo *et al*. [13] reported in a recent publication the dearth of data in suicidal behaviors in children and adolescent in low and middle-income countries within the African continent. They proposed a protocol for a systematic review determining the prevalence and risk factors of suicidal behavior in Sub-Saharian Africa's children and adolescents. Among the rare studies, a cross-sectional study among Malawi adolescents found a prevalence of 12.9% of suicidal attempts during a recall period of 12 months [14]. For suicidal ideation, a Ugandan study showed a rate of 23.5% in the past year [15]. In Tunisia, despite the particular attention that has been given to suicide in recent years, it remains, however, a subject of taboo in our society. This is reflected by the small number of studies directed on suicide, particularly among adolescents and the lack of national statistics [16]. To our knowledge, there is no study that has specifically examined the prevalence and associated factors of adolescents' suicidal thoughts and attempts in a Tunisian general population. It is this scarcity of data on suicidal behavior in Tunisia that prompted us to conduct this study among Tunisian adolescents a year after the revolution period. The aim of this study was to estimate the prevalence of suicidal thoughts and attempts among Tunisian high school students and to determine factors associated with these suicidal behaviors.

## Methods

**Participants:** this is a cross-sectional descriptive and analytical study. The population was composed of all high school students of the third grade of secondary school enrolled in the public and private secondary schools of the governorate of Monastir in Tunisia during the period of March and April in 2012. We decided to conduct our study on the third grade students because it represents relatively a stable period with less risk of bias. The population of high school students of the third grade contained 5674 students spread over 25 public high schools and 8 private schools. The sample size of the study was based on the following formula, for an error risk of 0.05 and a precision of 0.02:

$$N=\frac{\text{P}\times \text{Q}\times 4}{0.02^{2}}$$

With P being the prevalence of the phenomenon and set as 7.7% according to Lazreg *et al*. [17] and Q = 1 - P.

**Study design:** we opted for a cluster sampling by grouping the various institutions of the governorate into three groups of schools according to the total number of high school students in the third grade from each institution. The survey was administrated by a trained child and adolescent psychiatrist accompanied with one of the administrative staff of the school. We excluded from the study students that were absent on the day of the survey.

**Measures:** data collection was performed using a self-questionnaire that consisted of 4 parts: a pre-established form, the Beck Depression Scale [18], the Suicidal behavior Questionnaire-Revised (SBQ-R) [19], and the Rosenberg´s self-esteem scale [20]. The pre-established form contained 14 questions relating to socio-demographic characteristics, relationship with family members and peers, the existence of a history of maltreatment, personal and family medical history and life habits. School results were assessed by referring to the school evaluation of the previous academic year. Besides, the form contained 5 questions aimed at the detection of anxious manifestations inspired from the Hamilton anxiety scale [21]. The Beck Depression scale consisted of 21 items measuring the current level of the depressive symptomatology by quoting every item from 0 to 3. In case of presence of current depressive signs, the scale classifies the subjects into minor, mild and severe depression. To assess suicidal thoughts and attempts, we used the SBQ-R by Osman *et al*. [19] with author's permission. This questionnaire was translated into Arabic language and retranslated to English for language adaptation and the final version was considered after comparing the two versions. This instrument is made up of four items. The first item taps into lifetime suicide ideation and/or suicide attempt. The second item assesses the frequency of suicidal ideation over the past 12 months. The third item searches if the subject had spoken to somebody about his suicidal thoughts or intentions to commit suicide. Finally, the fourth item assesses self-reported likelihood of suicidal behavior in the future. We added to this questionnaire a multiple choice question investigating history of non suicidal self-injurious behavior. The self-esteem was assessed by the Rosenberg Self-Esteem Scale. The 10-item scale comprises four positively worded items and six negatively worded items, presented with the following response options: (1) strongly agree, (2) agree, (3) disagree, and (4) strongly disagree.

**Ethical issues:** written authorizations for the practice of the survey and the submission of the questionnaire were previously requested from the national and regional authorities. The ethics committee and the thesis committee of the Faculty of Medicine of Monastir have approved the protocol of this study. We provided the adolescents with our professional contact information and we expressed our will to help any person who requests medical care. The questionnaire was proposed to the students after a presentation of the study, and the participation was voluntary and anonymous. At the end of the auto-administrated form, the address and the phone number of a psychiatrist were transferred to the participants for consulting possibilities.

**Data analysis:** the data entry and statistical analysis were carried out by SPSS software (version 21.0 for Windows). Descriptive analysis included frequency calculation for qualitative variables, mean calculation and standard deviation for quantitative variables that have a normal distribution. Normality test was conducted by Kolmogorov-Smirnov test using a threshold of 0.05. The associations between the variables were studied by hypothesis tests in particular the Chi-square test and Fisher's exact test. After the univariate analysis, a logistic regression model was performed. The variables included in the model were the significant ones at the statistical threshold of 0.25 and the statistical significance was set at 5%. For these analytical investigations, suicidal behavior referred to lifetime suicide ideation and suicidal attempts were evaluated by the first item of the SBQ-R.

## Results

**Characteristics of the surveyed adolescents:** our study was sampled on a total of 821 adolescents including 560 girls and 261 boys resulting in a sex ratio of 0.46 (Table 1). The mean age of the students was 17.7 ± 0.97 years. The socioeconomic level was average in 46% of the adolescents. The study of the family status of the adolescents revealed that the majority (87.3%) lived in two-parent families. The relationship between the adolescents and their parents was described as very good in 40.1% of the cases, while only 7.3% of the population had a disturbed or very disturbed relationship. The relationship with other members of the family was good to very good in 95.7% of the adolescents. Regarding the relationship with peers, 94.8% of the students reported having friends. The relationship with peers was good to very good in 95.1% of the students surveyed. Concerning the school performance, 50.6% of the students surveyed had good to very good results, while only 9.3% had low scores. A pathological family history was reported by 28.5% of the adolescents which was divided into 22.5% of organic diseases, and 6% of psychiatric disorders. For the personal history, 23.6% of the adolescents had a history of a medical condition (chronic organic disease in 7.3%, and psychiatric illness in 16.3% of the cases). A history of child abuse was reported by 18.5% of the students. A history of non-suicidal self-injuries behavior was reported by 25.5% of the adolescents. In the search of regular use of psychoactive substances, it was reported that 10.9% of the students were smokers, 9.4% drank alcohol, and 5.3% used other substances, with cannabis (1.4%) being the most common. Concerning the current psychological characteristics, it was shown that 71.8% of the adolescents had depressive manifestations of varying intensity including 21.7% with mild depression and 25.3% reporting severe to very severe depression. For anxiety symptoms, more than half of our sample (55%) had anxiety manifestations. The self-esteem level assessed by the Rosenberg score was low among 17.3% of adolescents, medium among 75.3%, and high among 7.4% of them.

**Table 1:** Characteristics of the adolescents surveyed

Number		821
Age presented as mean (SD) years		17.7 (0.97)
Gender	Male	261 (31.8%)
	Female	560 (68.2%)
Socioeconomic status	Good	264 (32.1%)
	Average	377 (45.9%)
	Poor	180 (22%)
Type of family	Two-parent family	717 (87.3%)
	Single-parent family	104 (12.7%)
Relation with parents	Good to very good	758 (92.7%)
	Disturbed or very disturbed	60 (7.3%)
Relation with other members of the family	Good to very good	780 (95.7%)
	Disturbed or very disturbed	34 (4.1%)
Relation with peers	Good to very good	776 (95.1%)
	Poor	38(4.9%)
school performance	Good to very good	416 (50.6%)
	Average	329 (40.1%)
	Low	76 (9.3%)
Pathological family history	Somatic disorders	184 (22.5%)
	Psychiatric disorders	61 (6%)
Personal history	Chronic organic disease	60 (7.3 %)
	Psychiatric disorder	Depression (14.4%)
		Anxiety (1.2%)
		Other psychiatric disorders (0.7%)
	Non-suicidal self-injurious behavior	209 (25.5%)
	Child abuse	Sexual abuse: 15 (1.8%)
		Physical abuse: 88 (10.7%)
		Other types of abuse: 48 (5.8%)
Regular use of psychoactive substances	Smoking	88 (10.9%)
	Alcohol	76 (9.4%)
	Cannabis	11(1.4%)
	Other substances	32 (3.9%)
Current psychological characteristics	Depression	Mild: 177 (21.7%)
		Average: 202 (24.8%)
		Severe to very severe: 206 (25.3%)
	Anxiety	No anxiety symptoms: 451 (55%)
		Presence of anxiety: 370 (45%)
	Self-esteem	Low: 134 (17.3%)
		Medium: 582 (75.3%)
		High: 57 (7.4%)

**Figure 1:**
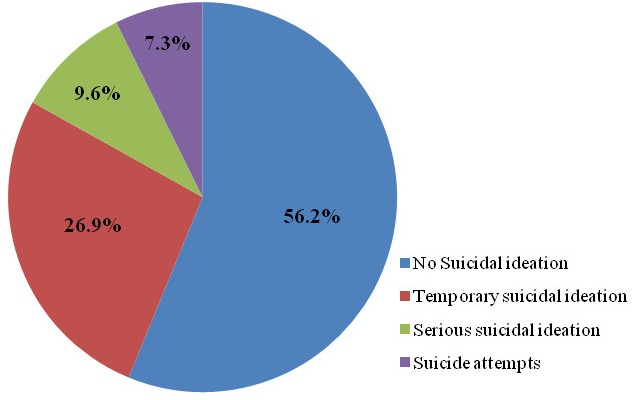
Prevalence of suicidal behavior in the adolescents surveyed

**Prevalence of suicidal behavior and associated factors:** the suicidal thoughts were absent in 56.2% of the students, while 26.9% of them reported brief passing suicidal ideation, and 9.6% reported serious suicidal ideation associated with suicidal planning (Figure 1). The suicide attempts were reported by 7.3% of adolescents. The frequency of suicidal ideation in the past 12 months was only once in 18.5% of adolescents. It was more than one time in 12.5% of adolescents, among these, 1.6% reported a frequency of five or more suicidal thoughts. Among all high school students, 25% of the teenagers have confessed their suicidal ideation to someone, while the remaining never acknowledged these thoughts. In addition, the intention of a future suicide attempt was declared by 7.8% of the surveyed adolescents. Among the students, 9.6% of the males, and 20.4% of females had suicidal behavior (Table 2), and the presence of suicidal behavior was significantly correlated with female gender (p< 0.001). When we looked into each type of suicidal behavior separately, we found that serious suicidal thoughts were reported by nearly three times more girls than boys (12.14% of girls versus 4.21% of boys, p = 0.001). Suicide attempts were also more frequent among girls (8.21% of girls versus 5.36% of boys) but the difference was not significant. Suicidal behavior was not associated with the family socioeconomic level (p = 0.35), nor the family type (p = 0.28). In addition, we did not find a statistically significant relationship between suicidal behavior and school performance (p = 0.29). By contrast, a disrupted relationship with the parents and/or with other members of the family was associated with the existence of suicidal behavior (p<0.001) and a same association between the relationships with peers and suicidal behavior was recorded (p = 0.001).

**Table 2:** The relationship between suicidal behavior and socio-demographic characteristics of the adolescents of the study

		Non suicidal behavior group (N=682)	With suicidal behavior group (N=139)	OR	CI 95%	*p*
**Gender**	Male	236 (90.4%)	25 (9.6%)	2.41	[1.52, 3.82]	< 0.001
	Female	446 (79.6%)	114 (20.4%)			
**Socioeconomic status**	Average to good	518 (82.7%)	108 (17.3%)	0.88	[0.56, 1.4]	0.35
	Poor	151 (84.4%)	28 (15.6%)			
**Type of family**	Tow-parents family	598 (83.5%)	118 (16.5%)	1.20	[0.71, 2.04]	0.28
	Single-parent family	84 (80.8%)	20 (19.2%)			
**Relationship with parents**	Good to very good	647 (85.4%)	111 (14.6%)	4.76	[2.76, 8.24]	<0.001
	Disturbed to very disturbed	33 (55%)	27 (45%)			
**Relationship with other members of the family**	Good to very good	657 (84.2%)	123 (15.8%)	3.73	[1.83, 7.60]	0.001
	Disturbed to very disturbed	20 (58.8%)	14 (41.2%)			
**Relationship with peers**	Good to very good	651 (83.9%)	125 (16.1%)	2.40	[1.18, 4.89]	0.016
	Poor	26 (68.4%)	12 (31.6%)			
**School performance**	Very good to average	621(91.1%)	124 (89.2%)	1.23	[0.67, 2.23]	0.29
	Low	61(8.9%)	15(10.8%)			
	Medium to high	562 (87.9%)	77 (12.1%)			

Suicidal behavior was more frequent among teenagers with a family medical history (p = 0.015) (Table 3). Concerning the type of the health problem, we could not establish any correlation between a family history of organic diseases and suicidal behavior (p = 0.37). However, adolescents with a family history of depression (p = 0.028) and other psychiatric disorders (p = 0.001) had more suicidal behavior than adolescents without a family history of psychiatric disorder. A relationship between suicidal behavior with a personal history of depression was established (p<0.001). This association was also valid for other psychiatric illnesses than depression (p = 0.007). Thus the presence of suicidal behavior was associated with the presence of a psychiatric personal history, whereas this could not be demonstrated for the organic medical history (p = 0.43). An association between suicidal behavior and the history of non-suicidal self-injurious behavior was detected (p<0.001). Adolescents with a history of sexual abuse reported more suicidal behavior (p<0.001), while the association between physical abuse and suicidal behavior was not significant (p = 0.056). We did not find any association between substances use and suicidal behavior (p=0.095 for smoking, p=0.21 for alcohol consumption, and p=0.54 for other substances consumption) in the univariate analysis. Current depressive symptoms assessed by Beck´s score were significantly associated with suicidal behavior (p<0.001). Similarly, the presence of anxiety symptoms had a statistically significant association with suicidal behavior (p<0.001). The relationship between low self-esteem and suicidal behavior was also statistically significant (p<0.001).

**Table 3:** The relationship between suicidal behavior and medical characteristics of the adolescents of the study

		Non suicidal behavior group	With suicidal behavior group			
Medical history		(N=682)	(N=139)	OR	CI 95%	*p*
**Pathological family history**	Pathological medical history	188 (78.3%)	52 (21.7%)	1.56	[1.06, 2.29]	0.015
	Somatic disorders	33 (17.9%)	151 (82.1%)	1.09	[0.71, 1.68]	0.37
	Depression	15 (65.2%)	8 (34.8%)	2.71	[1.12, 6.53]	0.028
	Other psychiatric disorders	17 (58.6%)	12 (41.4%)	3.69	[1.72, 7.93]	0.001
**Personal history**	Organic disease	49 (81.7%)	11 (18.3%)	0.90	[0.45, 1.78]	0.43
	Depression	72 (61%)	46 (39%)	4.19	[2.72, 6.43]	< 0.001
	Other psychiatric disorders	9 (56.3%)	7 (43.8%)	4.04	[1.47, 11.04]	0.007
	Child abuse	107 (67.7%)	51 (32.3%)	3.18	[2.12, 4.76]	< 0.001
	Physical abuse	64 (76.2%)	20 (23.8%)	1.62	[0.94, 2.78]	0.056
	Sexual abuse	6 (40%)	9 (60%)	7.80	[2.73, 22.28]	< 0.001
	Non suicidal self-injurious					
**Current psychological characteristics**	behaviors	133 (63.6%)	76 (36.4%)	4.97	[3.38, 7.29]	< 0.001
**Regular use of psychoactive substances**	Smoking	68 (77.3%)	20 (22.7%)	1.49	[0.87, 2.55]	0.095
	Alcohol	66 (86.8%)	10 (13.2%)	0.71	[0.35, 1.41]	0.21
	Cannabis and other substances	36 (83.7%)	7 (16.3%)	0.94	[0.41, 2.17]	0.54
**Depression**	Non depressive symptoms	224 (97.4%)	6 (2.6%)	10.87	[4.72, 25.03]	<0.001
	Presence of depressive symptoms	453 (77.4%)	132 (22.6%)			
**Anxiety**	Non anxiety symptoms	322 (91.7%)	29 (8.3%)	3.45	[2.24, 5.33]	<0.001
	With anxiety symptoms	326 (75.6%)	105 (24.4%)			
**Self-esteem**	Low	82 (61.2%)	52 (38.8%)	0.21	[0.14, 0.32]	<0.001
	Medium to high	562 (87.9%)	77 (12.1%)			

**Logistic regression analysis:** the multivariate regression analysis revealed six determining factors (Table 4): female gender (OR=2.56 (1.32-4.95); p=0.005), personal history of depression (OR=2.29 (1.38-3.80); p=0.001), tobacco smoking (OR=3.59 (1.61- 8.01); p = 0.002), current depressive symptoms (OR=5.50 (2.14-14.11); p<0.001), history of self-injurious behavior (OR=3.16 (2.05-4.86); p<0.001), and low self-esteem (OR=2.74 (1.71-4.38); p< 0.001).

**Table 4:** Baseline factors associated with suicidal behavior in the adolescents surveyed

Variables	Risk category	OR	95% CI	*p*-value
Gender (Ref. Male)	Female	2.563	1.327 – 4.951	0.005
Personnel history of depression (Ref. No)	Yes	2.297	1.389 – 3.800	0.001
History of non-suicidal self-injurious behaviors (Ref. No)	Yes	3.160	2.052 – 4.868	<0.001
Smoking (Ref. No)	Yes	3.596	1.614 – 8.015	0.002
Current depressive symptoms (Ref. No)	Yes	5.501	2.145 – 14.11	<0.001
Self-esteem (Ref. Good)	Low	2.741	1.714 – 4.384	<0.001

OR: Odds ratio, CI: Confidence Interval, Ref.: Reference category in the logistic regression

R^2^ = 0.24 and adjusted R^2^ = 0.37

## Discussion

Suicidal behavior of young population is a topic of extreme importance since suicide is irreversible, and should therefore be prevented. To our knowledge, this is the first study investigating the prevalence and factors associated with suicidal behavior among Tunisian adolescents. The sampling of this study was conducted in 2012, a year after the revolution with the aim of reassessment and examination of these results. A more recent comparative study on a similar population was conducted and the results are under evaluation. The prevalence of suicidal behavior varies across studies, depending on the terminology used and the methodology of investigation. Evans *et al*. [11] reported in a systematic review that the proportion of adolescents who revealed attempted suicide at least once in their lives was 9.7%, and 29.9% of adolescents claimed they had thought about suicide at some point in their lives. In addition, the prevalence of suicidal behavior varies across countries, depending on the culture, ethnicity, social, economic and religious characteristics of the population [22,23]. The frequency of suicidal behavior in Arab and Muslim countries is not well defined, given the scarcity of publications on this subject. This may be due to the fact that the subject of suicide remains a taboo, although less than before. In a study conducted on 805 Turkish school adolescents aged from 13 to 18 years using the Suicide Probability Scale, 2.5% of adolescents attempted suicide, at least once in their lives, and 23% thought, at least once, of committing suicide in the past 12 months [24]. In Tunisia, there is a scarcity of conducted studies on suicide in the general population and those interested in adolescents are even rare. A Tunisian study that was conducted with a sample of 685 students enrolled in public secondary schools in 1999 interested in behavior and needs for health found that 23.3% of the adolescents had suicidal thoughts and that 7.7% had already attempted suicide [17]. Our results on the prevalence of suicidal thoughts and suicide attempts are in line with this study. In our study, we found a female predominance for suicidal behavior. This result is in line with previously reported data indicating that the majority of suicide attempts were performed by females in Tunisian subpopulations [6,16]. This can be explained by the discrimination on the basis of gender [25,26]. In addition, intra-family conflicts were reported often as frequent triggers of suicide attempts in females [27], and Tunisian women are generally more exposed to all types of abuse than men [16]. On the international level, a similar trend for a gender imbalance in suicidal ideation and attempts has been also described [11]. The mortality by suicide is predominant in males, which can be explained by the use of more violent means of suicide [28].

In our sample, we did not find a relationship between the socio-economic level and suicidal behavior in adolescents, while other studies reported a positive correlation between this factor and suicidal behavior [28]. Generally low socioeconomic level is a trigger for stress and depressive symptoms, but also for low self-esteem and family relational difficulties which may reflect its role in suicidal behavior as reviewed earlier [29]. The role of family situation in the genesis of suicidal behavior is controversial. Some studies suggested that adolescents living in a single-parent family developed more suicidal behavior compared to children with both parents [30]. In contrast with these findings, the role of family structure in relation to suicidal behavior in adolescents was inconclusive and the results were contradictory [29]. In our population, the family status was not associated with suicidal behavior. According to the majority of studies, the quality of family dynamics is one of the determining factors in adolescent suicidal behavior [29]. In our study, the link between suicidal behavior and intra-familial and peers relationships could not be confirmed in the multivariate analysis. Several studies found a strong association between suicidal behavior, and peer bullying [31,32], while adolescents with good peer relationships had much lower suicide risk scores when they were compared to those with troubled relationships with peers [33]. In addition, we could not establish an association between bad school results and the existence of suicidal behavior as previously reported [12,33]. This observation can be explained by the fact that a minority of adolescents reported low school performance. Our study suggested an association between family psychiatric disorders history and suicidal behavior in the univariate analysis although this aspect was not confirmed by the multivariate study. These findings are consistent with some studies, where suicidal behavior was not correlated with having a psychiatric family history [33]. The association between personal history of mental disorders and suicidal behavior has been established by the majority of investigators regardless of gender [34-36]. Among these psychiatric disorders, emotional disorders and particularly depression were reported to be on top of the list of psychiatric illnesses that could lead to suicidal behavior [37]. Our study substantiates these findings since a personal background of depression was proven to be among the independent risk factors of suicidal behavior.

Considering history of child abuse, most studies reported that children who endured physical abuse are of a higher risk of developing suicidal behavior [38]. These results were established for both sexes and were valid for all ethnic groups [39]. Yet in our study, we found no significant association between physical abuse and suicidal behavior. The link between sexual abuse and suicidal behavior is more established for both genders [34, 40]. Many studies have shown that this relationship between sexual abuse and suicidal phenomena is direct [41,42], while others reported an indirect relationship mainly through depression, anxiety [43], and low self-esteem [44]. According to several authors, boys who were victims of sexual abuse are more likely than girls to engage in suicide attempts [45,46]. The non-association between sexual abuse and development of suicidal behavior in the multivariate analysis of this study can be explained by information bias. Undeniably, the number of adolescents who experienced sexual abuse during their childhood is greater in our sample. We think that some teenagers did not want to report such event despite the fact that our questionnaire was anonymous which can highlights the extent of the taboo that covers sexual abuse in our society. It was found in our sample that adolescents with a history of self-injury were three times more likely to have suicidal behavior than those who never engaged in self-injurious activities. This is in line with previous studies suggesting strong association between history of non suicidal self-injuries, and suicidal behavior [47]. In addition, self-injurious behavior is recognized a predictor of suicidal ideation and suicide attempts after controlling for other variables [48]. Some investigators suggested that self-harm can increase impulsivity and aggression leading to suicide attempts and completed suicide among non-suicidal individuals [49]. Smoking, alcohol consumption, and misuse of other psychoactive substances are correlated with suicidal attempts according to several studies [50,51]. For suicidal ideation, the results are more conflicting. While some studies suggested a positive relationship to suicidal behavior [52,53], others could not find any correlation [54]. The relationship of substance misuse to suicidal ideation is more important for boys than girls [55]. According to some studies the relationship between suicidal behavior and smoking is dose-dependent [56]. In our study, smoking was one of the determinant factors for suicidal behavior and this relationship was not found for other substances use. A minority of adolescents reported alcohol consumption and other substances. We believe that the consumption rates of these different substances are probably higher than they figure. Many teenagers may practice these habits in secret and find it difficult to reveal them especially in school.

The relationship between depression and suicidal behavior in adolescence was emphasized by numerous studies [28, 57,58]. In this study, suicidal behavior was positively associated with the presence of depression in the adolescent´s history, as well as the presence of current depressive symptoms. Suicidal ideation was positively correlated with depressive symptoms depending on their intensity [59]. This factor is considered more important for girls followed by the history of suicide attempts, while for boys the history of suicide attempts is first followed by depression [55, 60]. The presence of anxiety symptoms revealed a significant association with suicidal behavior. Pettit and collaborators showed that chronic stress and suicidal ideation are significantly associated even after controlling other psychiatric conditions [61] and stressful life events often precede suicidal behavior among young people [62]. We studied also the relationship between self-esteem and suicidal behavior. It has been confirmed by the majority of studies that poor self-esteem would be associated with a more important suicidal risk in adolescents [33], both for suicidal ideation and for suicide attempts [63,64]. Our results are in line with findings of the literature. The role of low self-esteem in suicidal behavior may be indirect by frequent association with depression [64], others common risk factors like homosexuality [65] and sexual abuse [44].

This study has several limitations. First, there were selection biases, we included only schooled teenagers and among them only those attending the third year. Thus our results cannot be generalized to the whole Tunisian adolescent population. Second, there is an information bias since we used a self-administrated questionnaire which is always a source of bias and the SBQ-R was not validated in the Tunisian cultural context.

## Conclusion

This study allowed to measure the prevalence of suicidal ideation and attempts in Tunisian adolescents and to identify associated factors that have a considerable interest in clinical and therapeutic fields. It will be interesting to generalize the study to all governorates in order to estimate the prevalence at the national level and improve the state of the knowledge about these behaviors. It is necessary after identifying risk factors to conduct interventional studies to explore the effectiveness of possible interventions before generalizing them. In addition, due to the specificity of the current political and economic conditions of Tunisia, conducting studies evaluating the evolution of suicidal behavior will be of great interest to study the impact of these changes on these behaviors. Several countries have put in place national strategies to combat suicide without encouraging results. The Tunisian government has set a technical committee with the objective to implement a strategy to prevent suicide. We think that our results can help to establish this strategy which required an idea about the prevalence and risk factors of suicidal behavior.

### What is known about this topic

- Depression is the most reported risk factor of suicide in adolescents;
- Suicidal behavior is frequent in adolescent clinical population in Tunisia.

### What this study adds

- This study confirmed the important prevalence of suicidal thoughts and attempts in Tunisian adolescents by assessing this prevalence in general population;
- In addition to depression, non suicidal self-injurious behaviors, smoking, low self- esteem, and female gender are revealed as independent associated factors to suicidal behavior.

## Competing interests

The authors declare no competing interests.

## References

[1] Breton JJ, Boyer R, Bilodeau H, Raymond S, Joubert N, Nantel MA (2002). Is evaluative research on youth suicide programs theory-driven? The Canadian experience. Suicide Life Threat Behav.

[2] Sawyer SM, Afifi RA, Bearinger LH, Blakemore SJ, Dick B, Ezeh AC (2012). Adolescence: a foundation for future health. Lancet.

[3] Steinberg L (2008). A Social Neuroscience Perspective on Adolescent Risk-Taking. Dev Rev.

[4] Liu XC, Chen H, Liu ZZ, Wang JY, Jia CX (2019). Prevalence of suicidal behaviour and associated factors in a large sample of Chinese adolescents. Epidemiol Psychiatr Sci.

[5] Shilubane HN, Ruiter RA, van den Borne B, Sewpaul R, James S, Reddy PS (2013). Suicide and related health risk behaviours among school learners in South Africa: results from the 2002 and 2008 national youth risk behaviour surveys. BMC Public Health.

[6] Halayem S, Bouden A, Othman S, Halayem MB (2010). Profil du suicidant en population clinique: une expérience tunisienne. Neuropsychiatrie de l'Enfance et de l'Adolescence.

[7] Nock MK, Borges G, Bromet EJ, Cha CB, Kessler RC, Lee S (2008). Suicide and suicidal behavior. Epidemiol Rev.

[8] Bonner RL, Rich AR (1987). Toward a predictive model of suicidal ideation and behavior: some preliminary data in college students. Suicide Life Threat Behav.

[9] Côté L, Pronovost J, Ross C (1990). Étude des tendances suicidaires chez des adolescents de niveau secondaire. Santé mentale au Québec.

[10] Im Y, Oh WO, Suk M (2017). Risk Factors for Suicide Ideation Among Adolescents: Five-Year National Data Analysis. Arch Psychiatr Nurs.

[11] Evans E, Hawton K, Rodham K, Deeks J (2005). The prevalence of suicidal phenomena in adolescents: a systematic review of population-based studies. Suicide Life Threat Behav.

[12] Chang HJ, Yang CY, Lin CR, Ku YL, Lee MB (2008). Determinants of suicidal ideation in Taiwanese urban adolescents. J Formos Med Assoc.

[13] Rukundo GZ, Kemigisha E, Ocan M, Adriko W, Akena DH (2018). A systematic review of the risk factors for suicidal ideation, suicidal attempt and completed suicide among children and adolescents in sub-Saharan Africa between 1986 and 2018: protocol for a systematic review of observational studies. Syst Rev.

[14] Shaikh MA, Lloyd J, Acquah E, Celedonia KL, M LW (2016). Suicide attempts and behavioral correlates among a nationally representative sample of school-attending adolescents in the Republic of Malawi. BMC Public Health.

[15] Culbreth R, Swahn MH, Ndetei D, Ametewee L, Kasirye R (2018). Suicidal Ideation among Youth Living in the Slums of Kampala, Uganda. Int J Environ Res Public Health.

[16] Majdoub W, Mosbahi A, Naouar M, Beji M, Mannai J, Turki E (2017). Suicide in children and adolescents: a Tunisian perspective from 2009 to 2015. Forensic Sci Med Pathol.

[17] Lazreg F, Ben Abdelaziz A, Gaha R, Ghedira A, Boussaadia A, Ghannem H (2005). Behaviours and needs for health of secondary-school-adolescents in Sousse (Tunisia)]). Tunis Med.

[18] Beck AT, Ward CH, Mendelson M, Mock J, Erbaugh J (1961). An inventory for measuring depression. Arch Gen Psychiatry.

[19] Osman A, Bagge CL, Gutierrez PM, Konick LC, Kopper BA, Barrios FX (2001). The Suicidal Behaviors Questionnaire-Revised (SBQ-R): validation with clinical and nonclinical samples. Assessment.

[20] Hensley WE, Roberts MK (1976). Dimensions of Rosenburg's self-esteem scale. Psychol Rep.

[21] Maier W, Buller R, Philipp M, Heuser I (1988). The Hamilton Anxiety Scale: reliability, validity and sensitivity to change in anxiety and depressive disorders. J Affect Disord.

[22] Nock MK (2009). Suicidal behavior among adolescents: correlates, confounds, and (the search for) causal mechanisms. J Am Acad Child Adolesc Psychiatry.

[23] Coskun M, Zoroglu S, Ghaziuddin N (2012). Suicide rates among Turkish and American youth: a cross-cultural comparison. Arch Suicide Res.

[24] Eskin M, Ertekin K, Dereboy C, Demirkiran F (2007). Risk factors for and protective factors against adolescent suicidal behavior in Turkey. Crisis.

[25] Kim WJ, Singh T (2004). Trends and dynamics of youth suicides in developing countries. Lancet.

[26] Petroni S, Patel V, Patton G (2015). Why is suicide the leading killer of older adolescent girls. Lancet.

[27] Akkaya-Kalayci T, Kapusta ND, Winkler D, Kothgassner OD, Popow C, Ozlu-Erkilic Z (2018). Triggers for attempted suicide in Istanbul youth, with special reference to their socio-demographic background. Int J Psychiatry Clin Pract.

[28] Hawton K, Saunders KE, O'Connor RC (2012). Self-harm and suicide in adolescents. Lancet.

[29] Evans E, Hawton K, Rodham K (2004). Factors associated with suicidal phenomena in adolescents: a systematic review of population-based studies. Clin Psychol Rev.

[30] Agerbo E, Nordentoft M, Mortensen PB (2002). Familial, psychiatric, and socioeconomic risk factors for suicide in young people: nested case-control study. BMJ.

[31] Kim YS, Leventhal BL, Koh YJ, Boyce WT (2009). Bullying increased suicide risk: prospective study of Korean adolescents. Arch Suicide Res.

[32] Brunstein Klomek A, Marrocco F, Kleinman M, Schonfeld IS, Gould MS (2007). Bullying, depression, and suicidality in adolescents. J Am Acad Child Adolesc Psychiatry.

[33] Engin E, Cuhadar D, Ozturk E (2012). Healthy life behaviors and suicide probability in university students. Arch Psychiatr Nurs.

[34] Shimshock CM, Williams RA, Sullivan BJ (2011). Suicidal thought in the adolescent: exploring the relationship between known risk factors and the presence of suicidal thought. J Child Adolesc Psychiatr Nurs.

[35] Patton GC, Harris R, Carlin JB, Hibbert ME, Coffey C, Schwartz M (1997). Adolescent suicidal behaviours: a population-based study of risk. Psychol Med.

[36] Reinherz HZ, Giaconia RM, Silverman AB, Friedman A, Pakiz B, Frost AK (1995). Early psychosocial risks for adolescent suicidal ideation and attempts. J Am Acad Child Adolesc Psychiatry.

[37] Brent DA, Johnson B, Bartle S, Bridge J, Rather C, Matta J (1993). Personality disorder, tendency to impulsive violence, and suicidal behavior in adolescents. J Am Acad Child Adolesc Psychiatry.

[38] Connor JJ, Rueter MA (2006). Parent-child relationships as systems of support or risk for adolescent suicidality. J Fam Psychol.

[39] Brodsky BS, Mann JJ, Stanley B, Tin A, Oquendo M, Birmaher B (2008). Familial transmission of suicidal behavior: factors mediating the relationship between childhood abuse and offspring suicide attempts. J Clin Psychiatry.

[40] Sorsdahl K, Stein DJ, Williams DR, Nock MK (2011). Associations between traumatic events and suicidal behavior in South Africa. J Nerv Ment Dis.

[41] Grossman DC, Milligan BC, Deyo RA (1991). Risk factors for suicide attempts among Navajo adolescents. Am J Public Health.

[42] Bensley LS, Spieker SJ, Van Eenwyk J, Schoder J (1999). Self-reported abuse history and adolescent problem behaviors II Alcohol and drug use. J Adolesc Health.

[43] Evans E, Hawton K, Rodham K (2005). Suicidal phenomena and abuse in adolescents: a review of epidemiological studies. Child Abuse Negl.

[44] Romans S, Martin J, Mullen P (1996). Women's self-esteem: a community study of women who report and do not report childhood sexual abuse. Br J Psychiatry.

[45] Garnefski N, Arends E (1998). Sexual abuse and adolescent maladjustment: differences between male and female victims. J Adolesc.

[46] Olshen E, McVeigh KH, Wunsch-Hitzig RA, Rickert VI (2007). Dating violence, sexual assault, and suicide attempts among urban teenagers. Arch Pediatr Adolesc Med.

[47] Evren C, Evren B (2005). Self-mutilation in substance-dependent patients and relationship with childhood abuse and neglect, alexithymia and temperament and character dimensions of personality. Drug Alcohol Depend.

[48] Toprak S, Cetin I, Guven T, Can G, Demircan C (2011). Self-harm, suicidal ideation and suicide attempts among college students. Psychiatry Res.

[49] Tang J, Yu Y, Wu Y, Du Y, Ma Y, Zhu H (2011). Association between non-suicidal self-injuries and suicide attempts in Chinese adolescents and college students: a cross-section study. PLoS One.

[50] Peltzer K, Pengpid S (2015). Early Substance Use Initiation and Suicide Ideation and Attempts among School-Aged Adolescents in Four Pacific Island Countries in Oceania. Int J Environ Res Public Health.

[51] Valdez-Santiago R, Solorzano EH, Iniguez MM, Burgos LA, Gomez Hernandez H, Martinez Gonzalez A (2018). Attempted suicide among adolescents in Mexico: prevalence and associated factors at the national level. Inj Prev.

[52] Lam TH, Stewart SM, Yip PS, Leung GM, Ho LM, Ho SY (2004). Suicidality and cultural values among Hong Kong adolescents. Soc Sci Med.

[53] Ali A, Maharajh HD (2005). Social predictors of suicidal behaviour in adolescents in Trinidad and Tobago. Soc Psychiatry Psychiatr Epidemiol.

[54] Riala K, Viilo K, Hakko H, Rasanen P (2007). Heavy daily smoking among under 18-year-old psychiatric inpatients is associated with increased risk for suicide attempts. Eur Psychiatry.

[55] Amitai M, Apter A (2012). Social aspects of suicidal behavior and preventio0n in early life: a review. Int J Environ Res Public Health.

[56] Breslau N, Schultz LR, Johnson EO, Peterson EL, Davis GC (2005). Smoking and the risk of suicidal behavior: a prospective study of a community sample. Arch Gen Psychiatry.

[57] Charfi F, Harbaoui A, Skhiri A, Abbes Z, Belhadj A, Halayem S (2019). [Epidemiological and clinical profile of suicide attempts in Tunisian children and adolescents after the revolution]. Pan Afr Med J.

[58] Liu X, Gentzler AL, Tepper P, Kiss E, Kothencne VO, Tamas Z (2006). Clinical features of depressed children and adolescents with various forms of suicidality. J Clin Psychiatry.

[59] Olvera RL (2001). Suicidal ideation in Hispanic and mixed-ancestry adolescents. Suicide Life Threat Behav.

[60] Pompili M, Mancinelli I, Girardi P, Ruberto A, Tatarelli R (2005). Childhood suicide: a major issue in pediatric health care. Issues Compr Pediatr Nurs.

[61] Pettit JW, Green KL, Grover KE, Schatte DJ, Morgan ST (2011). Domains of chronic stress and suicidal behaviors among inpatient adolescents. J Clin Child Adolesc Psychol.

[62] Shaffer D, Pfeffer CR (2001). Practice parameter for the assessment and treatment of children and adolescents with suicidal behavior. American Academy of Child and Adolescent Psychiatry. J Am Acad Child Adolesc Psychiatry.

[63] Overholser JC, Adams DM, Lehnert KL, Brinkman DC (1995). Self-esteem deficits and suicidal tendencies among adolescents. J Am Acad Child Adolesc Psychiatry.

[64] McGee R, Williams S (2000). Does low self-esteem predict health compromising behaviours among adolescents?. J Adolesc.

[65] van Heeringen C, Vincke J (2000). Suicidal acts and ideation in homosexual and bisexual young people: a study of prevalence and risk factors. Soc Psychiatry Psychiatr Epidemiol.

